# RNase MRP Cleaves Pre-tRNA^Ser-Met^ in the tRNA Maturation Pathway

**DOI:** 10.1371/journal.pone.0112488

**Published:** 2014-11-17

**Authors:** Yuichiro Saito, Jun Takeda, Kousuke Adachi, Yuko Nobe, Junya Kobayashi, Kouji Hirota, Douglas V. Oliveira, Masato Taoka, Toshiaki Isobe

**Affiliations:** 1 Department of Chemistry, Graduate School of Science and Engineering, Tokyo Metropolitan University, Tokyo, Japan; 2 Core Research for Evolutional Science and Technology (CREST), Japan Science and Technology Agency, Tokyo, Japan; 3 Division of Genome Repair Dynamics, Radiation Biology Center, Kyoto University, Kyoto, Japan; The John Curtin School of Medical Research, Australia

## Abstract

Ribonuclease mitochondrial RNA processing (RNase MRP) is a multifunctional ribonucleoprotein (RNP) complex that is involved in the maturation of various types of RNA including ribosomal RNA. RNase MRP consists of a potential catalytic RNA and several protein components, all of which are required for cell viability. We show here that the temperature-sensitive mutant of *rmp1*, the gene for a unique protein component of RNase MRP, accumulates the dimeric tRNA precursor, pre-tRNA^Ser-Met^. To examine whether RNase MRP mediates tRNA maturation, we purified the RNase MRP holoenzyme from the fission yeast *Schizosaccharomyces pombe* and found that the enzyme directly and selectively cleaves pre-tRNA^Ser-Met^, suggesting that RNase MRP participates in the maturation of specific tRNA *in vivo*. In addition, mass spectrometry–based ribonucleoproteomic analysis demonstrated that this RNase MRP consists of one RNA molecule and 11 protein components, including a previously unknown component Rpl701. Notably, limited nucleolysis of RNase MRP generated an active catalytic core consisting of partial *mrp1* RNA fragments, which constitute “Domain 1” in the secondary structure of RNase MRP, and 8 proteins. Thus, the present study provides new insight into the structure and function of RNase MRP.

## Introduction

Ribonuclease mitochondrial RNA processing (RNase MRP) is an essential eukaryotic ribonucleoprotein complex, generally consisting of one noncoding RNA (ncRNA) and several protein subunits [1]–[3]. Mutations in the human ncRNA cause a variety of recessive inherited disorders including cartilage-hair hypoplasia, which is characterized by short stature, hypoplastic hair, defective cellular immunity, and a predisposition to cancer [4]–[6], metaphyseal dysplasia without hypotrichosis [7], anauxetic dysplasia [8], kyphomelic dysplasia [9], and Omenn syndrome [10]. It has been reported that some inherited mutations in MRP RNA reduce the stability of the enzyme complex and/or alter its catalytic activity [11]–[14], but a mechanism linking the mutations to disease remains unknown.

The multisubunit composition of RNase MRP is remarkably similar to that of RNase P [1], [2], [15], [16]. In *Saccharomyces cerevisiae*, RNase MRP contains a 340 nt–long RNA component and ten essential proteins (listed in Table S1), eight of which are shared with RNase P [17]. RNase MRP has two additional subunits, Snm1 and Rmp1, which are not found in RNase P [18], [19]. Human RNase MRP and P also have similar subunit compositions (Table S1) [20]–[22].

The RNA component of RNase MRP is structurally related to that of RNase P [1], [23], [24]. However, the RNase P RNA is a catalytically active ribozyme [25]–[27], whereas the activity of RNase MRP RNA has not been reported [28]. RNase MRP RNA consists of two structural domains, termed Domain 1 and Domain 2 [1], [2], [29]. Domain 1 is believed to be a catalytic domain because the structure of this domain closely resembles that of RNase P and has major secondary structural elements conserved among RNase MRPs from a broad range of eukaryotes [29]–[31]. In addition, Domain 1 interacts with the protein subunits found in common with RNase P, including Pop1, Pop5, Pop6, Pop7, Pop8, and Rpp1 [23], [32]–[37]. On the other hand, Domain 2 appears to determine the enzyme's substrate specificity because the equivalent structure in RNase P serves to recognize pre-tRNA substrates [38]–[40]; interestingly, the Domain 2 sequence is **not** similar to the corresponding sequence of RNase P [1], [2], [24]. Although Esakova *et al.* recently reported that *S. cerevisiae* RNase MRP binds the substrate with Domains 1 and 2 *in vitro* [41], the structural elements that define the catalytic activity and substrate specificity of RNase MRP remain largely unknown.

RNase MRP has different cellular substrates than RNase P. Whereas RNase P cleaves primarily tRNAs and participates in tRNA maturation [3], [42], [43], RNase MRP targets (i) the site A_3_ of the internal transcribed spacer 1 (ITS1) between 18S and 5.8S ribosomal RNAs (rRNAs) in the precursor 27SA2 rRNA during ribosome biogenesis in the nucleolus [44], [45], (ii) a subset of mRNAs involved in cell-cycle regulation [46]–[48], and (iii) other RNAs including a certain type of mRNA, snoRNA, transposon RNA, and viral RNA [48]–[50]. It has also been reported that a dimeric tRNA precursor, pre-tRNA^Ser-Met^, might be a substrate of RNase MRP [51] because a pre-tRNA intermediate accumulates in a *Schizosaccharomyces pombe* mutant defective for RNase MRP. tRNA maturation requires cleavage of the dimeric pre-tRNA^Ser-Met^, which generates pre-tRNA^Ser^ having a 5′ leader sequence, intron, and the 3′ “trailer” sequence, and pre-tRNA^Met^ having a mature 5′ end and 3′ trailer sequence (Figure S1) [52]. However, direct experimental evidence that RNase MRP participates in this process has not been obtained.

To elucidate the role of RNase MRP in tRNA processing, we prepared a temperature-sensitive (*ts*) *S. pombe* mutant of *rmp1*, a unique protein component of RNase MRP, and analyzed the phenotype of this mutant. We also purified RNase MRP from *S. pombe* and directly examined its catalytic activity. Based on our results, we propose that RNase MRP is responsible for the maturation of pre-tRNA^Ser-Met^. We also present results for limited nucleolysis of purified RNase MRP and show that *mrp1* is the RNA component of *S. pombe* RNase MRP and that Domain 1, in the context of the holoenzyme, is responsible for the catalytic activity of this multisubunit enzyme complex.

## Results

### Inactivation of RNase MRP causes the accumulation of pre-tRNA^Ser-Met^

**Figure 1:**
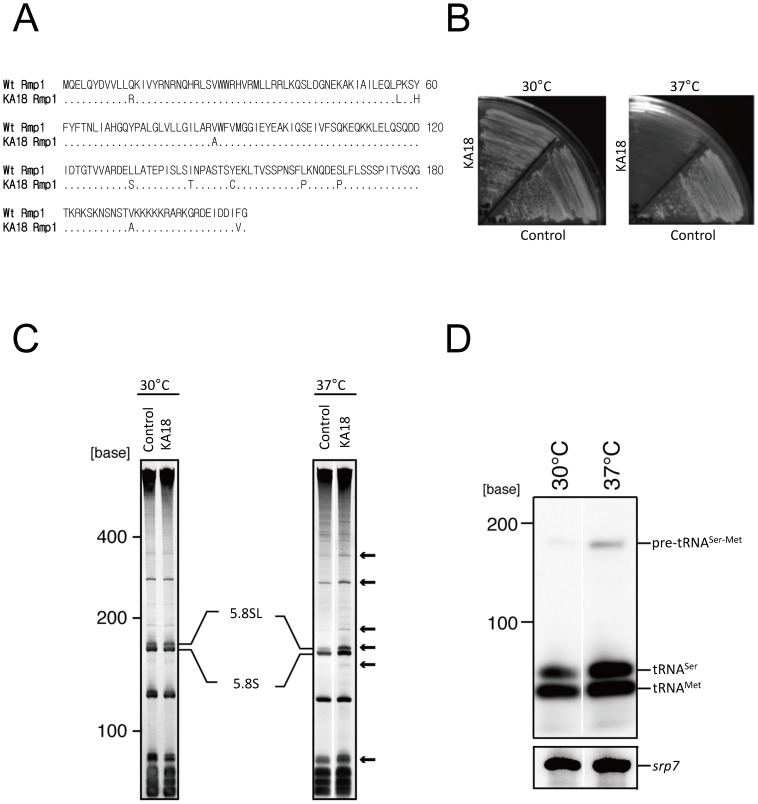
Pre-tRNA^Ser-Met^ accumulates in the KA18 *ts rmp1* mutant. (A) Rmp1 mutations in yeast strain KA18. The 11 amino acid substitutions in Rmp1 of KA18 are indicated in the figure. (B) KA18 and the control strain (KA13, Table S6) were spread onto YES plates and incubated at 30°C or 37°C for 3 days. (C) Analysis of RNAs in KA18 and KA13 cells grown at 30°C or 37°C. RNAs extracted from cells after incubation for 20 h at the indicated temperature were separated on 8 M urea-7.5% polyacrylamide gels and visualized with SYBR Gold staining. Arrows indicate RNAs that accumulated in KA18 as compared with KA13. (D) Northern blot analysis of pre-tRNA^Ser-Met^. The analysis was performed after incubation for 20 h at each indicated temperature. The *srp7* RNA was used as a loading control [87], [88].

Because all the components of RNase MRP are essential for cell viability [1], [2], the cellular role of this enzyme has been studied mainly using *ts* mutants carrying mutations in the gene for *mrp1* RNA [51]–[54], Rmp1 [19] or Snm1 protein [55]. We tried to isolate a fission yeast (*S. pombe*) *ts* mutant caused by mutation in Rmp1, a protein subunit specific to RNase MRP. By screening yeast strains carrying mutations in Rmp1, we obtained a *ts* strain, termed KA18, that carries mutations in Rmp1 that result in 11 amino acid substitutions: Q12R, P57L, Y60H, V86A, L132S, I142T, Y149C, L161P, S167P, V192A, and F210L (Figure 1A). Interestingly, we found that none of those mutations corresponded to that of the *ts S. cerevisiae* mutant of Rmp1, which had a single amino acid substitution of Cys-103 (Leu-80 in S. pombe Rmp1) to Arg [19]. KA18 exhibited a severe growth retardation phenotype under the nonpermissive temperature (37°C) (Figure 1B). When KA18 cells were grown at 37°C, several RNAs accumulated to abnormal levels as compared with the control strain (Figure 1C). In particular, KA18 exhibited a 6-fold increase in the level of the long form of the 5.8S (5.8SL) rRNA compared with the wild-type strain. This is consistent with previous reports that 5.8SL rRNA accumulates in the *ts* strain that has a mutation in *mrp1* RNA or Rmp1/Snm1 protein owing to the reduced cellular activity of RNase MRP to cleave site A_3_ [19], [53]–[56], indicating that KA18 has a defect in RNase MRP activity.

To examine whether RNase MRP is involved in tRNA^Ser^ and tRNA^Met^ maturation [51], we analyzed the level of pre-tRNA^Ser-Met^ in KA18 cells by Northern blotting. As shown in Figure 1D, pre-tRNA^Ser-Met^ accumulated to an abnormal level in KA18 grown at 37°C, whereas the cellular level of *srp7* (control RNA) remained unchanged in KA18 cells (Figure 1D), suggesting that RNase MRP cleaves pre-tRNA^Ser-Met^ *in vivo*.

### RNA and protein components of *S. pombe* RNase MRP

**Figure 2:**
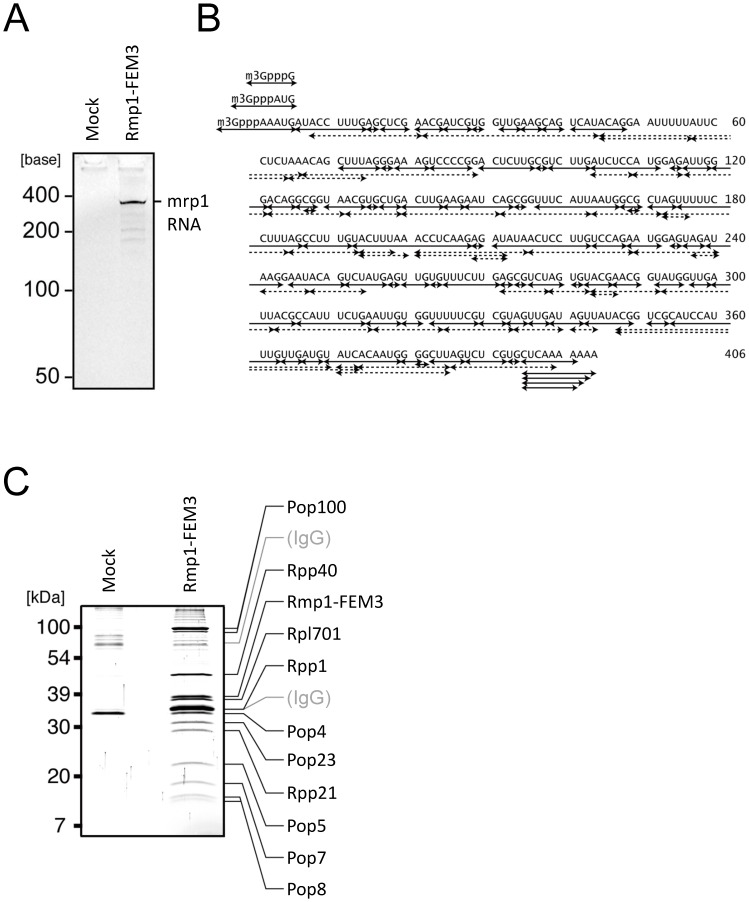
Components of the *S. pombe* RNase MRP complex. (A) RNA component of *S. pombe* RNase MRP (*mrp1*). RNA was separated from the purified RNase MRP with acid-phenol treatment and subjected to 8 M urea-7.5% PAGE (SYBR Gold staining). (B) Nucleotide sequence of *S. pombe mrp1* RNA and the fragments used for the sequence analysis. Solid or dashed double-headed arrows show the fragments obtained by digestion with RNase T1 or MazF/PemK RNase, respectively. RNase T1 fragments were identified by Ariadne search program, and PemK/MazF fragments were identified by manual inspection of MS/MS spectra (see also Table S2). m_3_Gppp, trimethylguanosine cap. (C) Protein components of *S. pombe* RNase MRP. The RNase-MRP preparation affinity-purified using FEM3-tagged Rmp1 as bait (Rmp1-FEM3) was separated by SDS-PAGE and visualized with Coomassie Brilliant Blue staining. The proteins identified by LC-MS/MS are shown on the right (see also Table S3 and Figure S6). The IgG probably resulted from sloughing from the beads during the affinity purification.

Previous studies showed that the catalytically active RNase MRP isolated from yeast *S. cerevisiae* and from human HEp-2 cells consists of a single ncRNA of 340 and 277 nt and 9 and 10 protein components, respectively [17]–[22]. To isolate the *S. pombe* RNase MRP, we employed tandem affinity purification using Rmp1 fused with a FEM-3 tag (FLAG, TEV cutting site, and 3× Myc attached to the C-terminus) as bait. The resulting complex was catalytically active against the known substrate of RNase MRP, ITS1 RNA (Figure S2). This RNase MRP preparation contained a single major RNA of ∼400 nt, the predicted size of *S. pombe mrp1* RNA from the size of *S. cerevisiae* RNase MRP RNA (Figure 2A). This RNA band was excised from the PAGE gel, digested with RNase T1 or with MazF/PemK RNase, and the fragments were analyzed by tandem mass spectrometry (MS/MS) coupled with a genome-oriented search engine Ariadne [57]. The analysis identified all fragments covering the total sequence of *mrp1* RNA (Figure 2B and Table S2). In addition, we found that the *S. pombe mrp1* RNA had heterogeneous 5′-terminal sequences, AAAUG, AUG and G, each with a 5′-trimethylguanosine cap (Figure S3). This cap structure indicates that the *mrp1* RNA is transcribed by RNA polymerase II, as noted for *S. cerevisiae nme1* RNA [58]. We also found that the RNA had heterogeneous 3′-terminal sequences, CUCAAAG-OH and an additional one to four adenines at the 3′-end in place of G (CUCAAAA_1–4_-OH, Figure 2B and Table S2). This supports the previous reports that the primary transcript of *mrp1* is processed by an exonuclease that catalyzes 3′-trimming during the biogenesis of RNase MRP and adenines were added later [59], [60]. However, the biological significance of this heterogeneity is obscure.

The proteomic analysis of the *S. pombe* RNase MRP by SDS-PAGE and tandem MS identified 11 protein components (Figure 2C and Table S3, see also Nomenclature in Materials and Methods). The identified proteins included all 10 components of *S. pombe* RNase MRP predicted in Pombase (http://www.pombase.org/), indicating that our RNase MRP preparation was typical of those described previously. Our preparation, however, contained one additional protein subunit, Rpl701, which had not been identified in RNase MRP of any organisms studied [1]–[3]. Rpl701, generally known as subunit L7 of the large ribosome, was reproducibly detected in the RNase MRP complex prepared multiple times. Furthermore, the reverse pull-down analysis using a tagged Rpl701 as bait allowed isolation of RNase MRP from *S. pombe* cells, whereas tagged Rpl702 or Rpl703, the paralogs of Rpl701, failed to recover the enzyme complex (Figure S4). Thus, we concluded that Rpl701 is a novel component of RNase MRP in *S. pombe*. According to the image analysis of the SDS-PAGE profile, *S. pombe* RNase MRP complex consisted of single copies of each protein subunit, including Rpl701, except for Rpp1, which was present at two copies per complex (Table S4).

### RNase MRP cleaves pre-tRNA^Ser-Met^ *in vitro*

**Figure 3:**
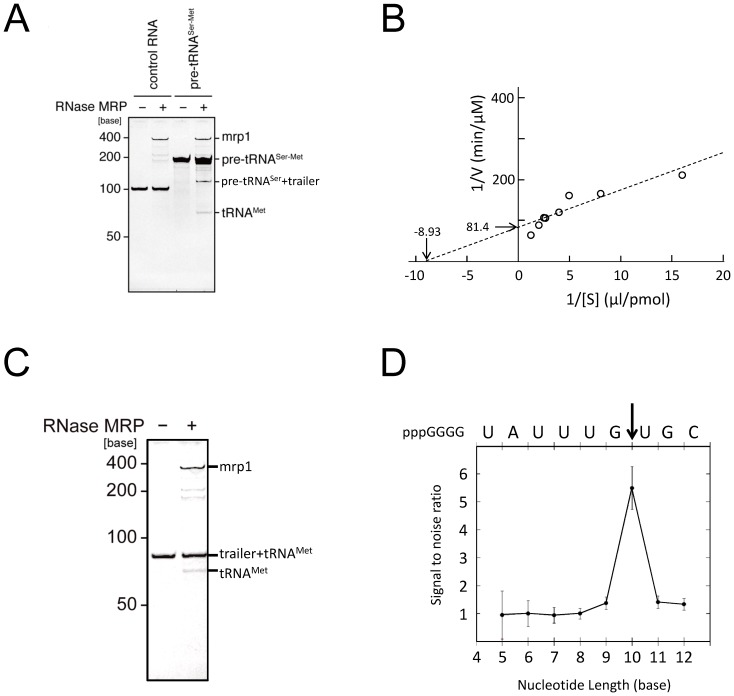
Purified *S. pombe* RNase MRP cleaves pre-tRNA^Ser-Met^. (A) *In vitro* cleavage assay of dimeric pre-tRNA^Ser-Met^. Purified RNase MRP (1 pmol) was incubated with pre-tRNA^Ser-Met^ (8 pmol) or with a control RNA (pre-tRNA^Ser^, 8 pmol) at 37°C for 30 min and subjected to 8 M urea-7.5% PAGE (SYBR Gold staining). Note that RNase MRP cleaves pre-tRNA^Ser-Met^ into two major RNAs, “pre-tRNASer + trailer” sequence and tRNA^Met^ (see Figure S1 for details). (B) Double-reciprocal plot of the catalytic reaction mediated by RNase MRP. The reaction was performed for 15 min with synthetic pre-tRNA^Ser-Met^ as a substrate. The V was calculated from the quantity of pre-tRNA^Ser^, which was estimated from the intensity of the pre-tRNA^Ser^ band after PAGE of the reaction mixture. The plot indicates K_M_ of 0.112 µM and Vmax of 12.3 nmol/min. (C) RNase MRP cleavage of trailer+tRNA^Met^. The reaction was performed under the conditions described in (A), and the product was analyzed with 8 M urea-7.5% PAGE (SYBR Gold staining). (D) Identification of the cleavage site between the trailer sequence and RNA^Met^. The reaction product obtained in (C) was analyzed directly by LC-MS. The ion-peak intensities of the 5′ fragments from 5- to 12-nt lengths were plotted. The values represent the mean ± standard deviation of three independent assays. Letters above the profile indicate the 5′ sequence of trailer+tRNA^Met^. An arrow indicates the cleavage site of the reaction. Note that RNase MRP produced tRNA^Met^ with a mature 5′-sequence [89].

To examine whether RNase MRP directly cleaves the dimeric precursor tRNA to promote tRNA maturation, we performed *in vitro* cleavage analysis. The purified RNase MRP pulled down with a tagged Rmp1 cleaved *in vitro*-transcribed pre-tRNA^Ser-Met^ into two RNA fragments under the experimental conditions employed, although it was not reactive to pre-tRNA^Ser^ used for a control RNA (Figure 3A). Kinetic analysis of this reaction estimated a Michaelis constant (K_M_) of 0.112 µM and Vmax of 12.9 nM/min (Figure 3B).

To determine the cleavage site, we prepared a synthetic substrate, “trailer”+tRNA^Met^ (Figure S1), digested it with the purified RNase MRP, and analyzed the products by SDS-PAGE and liquid chromatography (LC)-MS/MS. The PAGE analysis detected a single RNA product at a position corresponding to the size of mature tRNA^Met^ (Figure 3C). The LC-MS analysis detected a nucleolytic fragment pppGGGGUAUUUUG derived from the “trailer” sequence (Figure 3D) and produced a 5′ end of mature tRNA^Met^. We also found that the fragment pppGGGGUAUUUUG has a hydroxyl group at 3′ terminus, consistent with the reported cleavage specificity of RNase MRP [61].

### The RNase-resistant core RNP of MRP cleaves pre-tRNA^Ser-Met^

**Figure 4:**
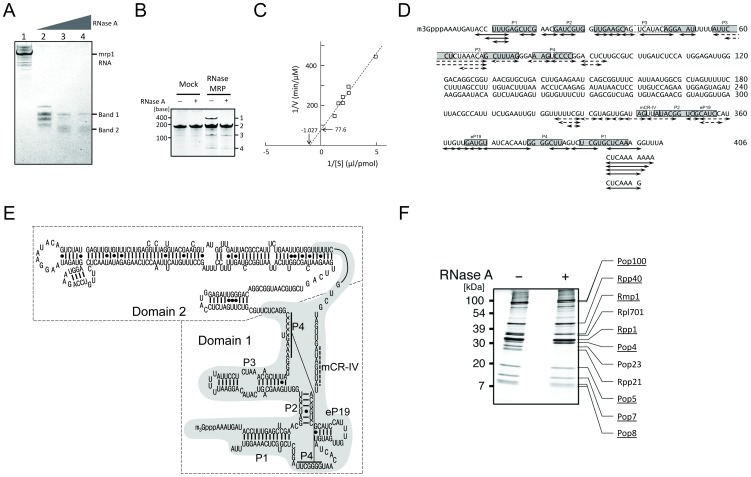
Isolation of the catalytically active core of RNase MRP. (A) Urea-PAGE profiles of *mrp1* RNA fragments produced by RNase A–mediated limited nucleolysis of RNase MRP (SYBR Gold staining). The *S. pombe* RNase MRP obtained from a 2-l culture of logarithmically growing cells was digested on FLAG M2 agarose beads at 4°C for 1 h with increasing amounts of RNase A. Lane 1, no RNase A; lane 2, 1 µg/ml; lane 3, 5 µg/ml; lane 4, 10 µg/ml. A portion (5%) of each reaction was loaded per lane. (B) A core of RNase MRP produced by RNase A–mediated limited nucleolysis cleaves pre-tRNA^Ser-Met^. RNase MRP (pulled down with tagged Rmp1 from JJ095 cells using FLAG M2 agarose) and the mock preparation (pulled down from SP6 cells) were incubated with (+) or without (−) RNase A. Each digested preparation (1 pmol each) was incubated with pre-tRNA^Ser-Met^ (8 pmol) at 37°C for 30 min, and each reaction mixture was subjected to urea-PAGE (SYBR-Gold staining). Band 1, *mrp1* RNA; 2, pre-tRNA^Ser-Met^; 3, pre-tRNA^Ser^+trailer; 4, tRNA^Met^. (C) Double-reciprocal plot of the catalytic reaction of RNase A–mediated partial nucleolysis of RNase MRP. The reaction was performed for 30 min with synthetic pre-tRNA^Ser-Met^ as a substrate under the conditions as in Figure 3B. The plot indicates K_M_ of 0.974 µM and Vmax of 12.9 nM/min. (D) RNA fragments produced by RNase T1 digestion of Band 1 (indicated by solid lines) and Band 2 (broken lines) in (A). The fragments identified by LC-MS/MS are mapped on the *mrp1* RNA sequence, where the conserved helices and strands of *mrp1* [1], [29], [31] are shown in shaded boxes. (E) Nuclease-resistant regions mapped in the secondary structure of *mrp1*. The map was according to the previous study [2] with modifications made by the assistance of CentroidHomFold (http://www.ncrna.org/centroidhomfold). Dashed-line boxes denote the two putative domains [2]. The nuclease-resistant region is shaded gray. The nucleotides with dotted bar are the consensus sequence ANAGNNA known as the mCR-IV motif [2]. (F) SDS-PAGE profiles of the protein components of RNase MRP before (−) and after (+) RNase A–mediated limited nucleolysis (Coomassie Brilliant Blue staining). The proteins assigned by LC-MS/MS are also shown. Note that the catalytic core of RNase MRP produced by partial nucleolysis contains 8 (underlined) of the 11 total subunits.

To determine the structural elements necessary for the catalytic activity of RNase MRP, we performed limited nucleolysis of our RNase MRP preparation using RNase A. Although the *mrp1* RNA was gradually degraded into smaller fragments by digestion with increasing RNase A concentrations at 4°C, we found two SYBR Gold–stained bands that contained relatively stable RNA fragments with approximate sizes of 150 and 120 nt (assigned as Band 1 and Band 2 in Figure 4A). We recovered the ribonucleoprotein complex of this partial nucleolysis and examined its catalytic activity using pre-tRNA^Ser-Met^ as a substrate. As shown in Figure 4B, this RNase A–treated MRP preparation retained the ability to cleave pre-tRNA^Ser-Met^ (Figure 4B). Kinetic analysis estimated a K_M_ of 0.974 µM and Vmax of 12.3 nM/min for the reaction mediated by this catalytic core (Figure 4C). Although this K_M_ value is ∼10 times greater than that estimated for the intact RNase MRP, the Vmax compares with that estimated for the intact MRP (12.9 nM/min, Figure 3B), suggesting that the limited RNase A cleavage produced an active degradation intermediate of RNase MRP with reduced affinity for the substrate RNA. We found, however, this RNase MRP intermediate did not cleave ITS1 substrate (Figure S5; see Discussion).

To characterize this partially degraded MRP complex, Bands 1 and 2 in Figure 4A were excised from a PAGE gel, in-gel digested with RNase T1, and analyzed by tandem MS; the analysis identified 24 RNA fragments for Band 1 and 18 fragments for Band 2 (Figure 4D). Mapping these fragments on the *mrp1* sequence showed that they covered 100–150 nt in the 5′ and 3′ terminal regions of the *mrp1* RNA. Interestingly, most of the fragments were from Domain 1 of the *mrp1* secondary structure (Figure 4E). To exclude the possibility that any small RNA fragments from Domain 2 might have nucleolytic activity, we performed direct LC-MS analysis of the RNase A–treated MRP RNAs without PAGE separation. We found only a small population of RNA fragments mapped on Domain 2 (<2% of total RNA identified); (Table S5), demonstrating that the active catalytic core of RNase MRP produced by RNase A–mediated partial nucleolysis consisted of RNA fragments that are almost exclusively located in Domain 1.

We also analyzed the protein components of the active MRP core complex. The proteomic LC-MS analysis identified 8 of 11 protein subunits, whereas 3 subunits, Pop23, Rpp21, and Rpl701, were absent (Figure 4F). We estimated that the stoichiometry of the 8 subunits associated to the core complex remained essentially the same as in the intact enzyme (Table S4), suggesting that these subunits are tightly associated with each other and with Domain 1 of the *mrp1* RNA to constitute an active catalytic core of the RNase MRP complex.

## Discussion

Our K_M_ value of 112 nM for RNase MRP–mediated cleavage of tRNA^Ser-Met^ *in vitro* compares well with those estimated for the catalytic reaction of tRNA precursors mediated by RNase P from various sources; *i.e*., 20–240 nM for RNase Ps in *S. pombe* [62], *S. cerevisiae* [63], *Dictyostelium discoideum* [64], and in *Drosophila melanogaster* [65]. In addition, it has been reported that the cellular concentration of RNase MRP is similar to that of RNase P [2] and that most RNase MRP localizes primarily in nucleoli [66], [67], where pre-tRNAs exist [68]. Based on these observations, we propose that RNase MRP participates in the processing of particular pre-tRNAs in collaboration with RNase P.

Our purified RNase MRP preparation cleaved a synthetic substrate, trailer+tRNA^Met^, and produced a “trailer” nucleotide with 3′-OH and tRNA^Met^ with a 5′-phosphate (Figure 3D). This is consistent with the cleavage specificity reported for RNase MRP. Regarding the sequence specificity of the cleavage, there is an argument that this enzyme cleaves at the 5′ position of the fourth nucleotide from a cytosine [69] or has a broader specificity [54]. In our experiment, the enzyme cleaved a G-U bond in a “trailer” sequence (Figure 3D), suggesting that RNase MRP has rather broad cleavage specificity that certainly requires further investigation.

Several research groups have studied RNase MRP mainly by mutational analysis of the RNA component, and the structure/function relationship of this multisubunit enzyme has been reported [19], [53]–[56]. In this study, we produced a core of RNase MRP by partial nucleolysis and showed its nuclease activity (Figure 4B and 4C). From the analysis of the constituents of this catalytic core, we propose that the RNP complex of Domain 1 *mrp1* RNA, which associates with eight protein subunits (Pops4, 5, 7, 8, and 100, Rmp1, Rpp1, and Rpp40), is responsible for the catalytic activity of RNase MRP. Another structural element, Domain 2 *mrp1* RNA and three protein subunits, Pop23, Rpp21, and Rpl701, may have a role in stabilizing the enzyme/substrate complex and thereby determining substrate specificity. Thus, RNase MRP has a molecular architecture similar to that of RNase P (Table S1), which is composed of a catalytically active RNA domain and a structural element important for stable binding to substrate tRNAs [70], [71]. Namely, Domain 2 and its associated protein subunits in RNase P constitute a “specificity domain”, which has a role in the recognition of the TΨC stem–loop of the substrate pre-tRNA and can bind to a proper position of the substrate, thus conferring the specificity for pre-tRNA substrates [38]–[40], [72]–[74].

Our study identified a novel protein subunit, namely Rpl701, of fission yeast RNase MRP. Rpl701 is probably a cofactor of the Domain 2 RNP complex because it was not detected in the Domain 1–associated catalytic core (Figures 4F). Although Rpl701 is not found in *S. cerevisiae* or human RNase MRP (Table S1), recent studies identified a *S. cerevisiae* homolog of Rpl701 as a protein factor required to construct a proper pre-rRNP structure for accurate A_3_ pre-rRNA processing [75], [76]; in particular, Rpl701 is a *trans*-acting factor in *S. cerevisiae,* which potentially recruits RNase MRP to the A_3_ site of rRNA or removes the enzyme from the A_3_ site after the processing reaction [77]. Our observation that the RNase-resistant core of RNase MRP lacking Rpl701 did not cleave ITS1 substrate (Figure S5) also suggests that Rpl701 acts as a *trans*-acting factor rather than a component necessary for the catalytic activity in *S. pombe* RNase MRP. Thus, it might be possible that fission yeast incorporated this *trans*-acting factor into the functional enzyme complex during evolution, presumably to improve the efficiency of ribosome biogenesis. Regarding this point, it is interesting to note that the function of Rpl701 could not be replaced by Rpl702 or Rpl703, which has high sequence similarity to Rpl701 (87% or 55% identity, respectively).

## Materials and Methods

### Yeast strains, media, and culture

Table S6 lists the *S. pombe* strains used in this study. General genetic procedures were carried out as described [78]. Standard rich yeast extract medium supplemented with leucine (YES) and Edinburgh minimal medium were used. G418 antibiotic was purchased from Nacalai Tesque.

### Nomenclature

Because the specific gene names of RNase MRP components have not been finalized for *S. pombe*, we defined them as in Table S1. The nomenclature was according to the sequence similarity of the protein product in *S. pombe* to the equivalent product in *S. cerevisiae* or *Homo sapiens*.

### Construction of plasmids and transformants for tagged-protein expression

The details for the targeting and expression vectors used in this study have been archived in GenBank. pCtFEM3ki-spac323.08-kanMX6T (accession no. AB623236), containing the gene *kanMX6* as a marker, was used as the targeting vector to make the JJ095 strain for purification of the MRP RNase complex. SP6 cells were transformed with the resulting vector as described [79]. To screen for *kanMX6*-carrying transformants, cells were spread on YES plates containing 0.1 mg/ml G418.

For constitutive expression of HATA (HA, TEV cutting site, protein A)-tagged ribosomal proteins Rpl701, Rpl702, and Rpl703 and the tag without protein, pFOX1-rpl701-HATA (AB623239), pFOX1-rpl702-HATA (AB623240), pFOX1-rpl703-HATA (AB623241), and pFOX1-CHATA (AB623238) were used as expression vectors, respectively. The JJ095 cells (Table S6) were transformed with each vector and spread on Edinburgh minimal medium plates to screen for *leu2* carrying the transformants.

### Random mutagenesis to establish *ts rmp1* mutants

The coding DNA of *rmp1* (*spac323.08*) containing mutations was generated by PCR amplification of *S. pombe* genomic DNA using primers Eco-SPAC323.08-F and Nde-SPAC323.08-R (Table S7) and the nucleotide analog procedure (JBS dNTP-Mutagenesis Kit, JENA Bioscience). The mutagenized DNA was integrated into the EcoRI-NdeI site of vector pCtFLATAki-kanMX6 (AB623235). In addition, the 3′ noncoding sequence of *rmp1* was amplified by PCR using primers RV-Tspac323.08-F and Sph-Tspac323.08-R (Table S7) and then integrated into the EcoRV-SphI site of the same vector. To replace chromosomal *rmp1* with a mutant allele, the plasmid was transfected into SP6 cells as described [79]. G418-resistant transformants were obtained from YES plates. To select the *ts* clones, the transformants were replicated onto YES plates and separately incubated at a permissive temperature (30°C) and at the nonpermissive temperature (37°C). Clones that could not grow at 37°C were considered as *ts* mutants for RNase MRP, and their chromosomal *rmp1* DNAs were sequenced.

### Northern blotting

Total RNA was extracted from *S. pombe* cells according to the method described [80]. Northern blotting was performed using a DIG RNA labeling kit (SP6/T7) and a DIG luminescent detection kit (Roche Applied Science). The template DNAs including the T7 promoter for synthesizing RNA probes to detect precursor and mature tRNAs and *srp7* were amplified by PCR from *S. pombe* genomic DNA using the primers listed in Table S7.

### Purification of the intact RNase MRP complex

Intact RNase MRP was purified as described [81] with modifications. Cells constitutively expressing FEM-3-tagged Rmp1 (JJ095) were collected from a 2-l culture by centrifugation and suspended in an equal volume of lysis buffer (50 mM HEPES, pH 7.6, 300 mM potassium acetate, 5 mM magnesium acetate, 20 mM β-glycerol phosphate, 1 mM EGTA, 1 mM EDTA, 0.1% (v/v) Nonidet P-40, 1 mM DTT, 1 mM PMSF, and a protease inhibitor cocktail (Sigma). The suspension was frozen in liquid N_2_ and homogenized using a Multi-beads shocker (Yasui Kikai Co. Ltd). After removal of the debris by centrifugation at 100,000×*g* for 30 min at 4°C, the extracts were incubated with anti-myc IgG (9E10) conjugated to agarose (sc-40 AC, Santa Cruz Biotechnology) at 4°C for 2 h. The precipitates were washed with wash buffer (50 mM HEPES, pH 7.4, 150 mM NaCl, 0.25% [v/v] NP-40) and treated with the AcTEV protease–containing buffer (50 mM HEPES, pH 7.4, 150 mM NaCl, 0.25% [v/v] NP-40, 1 mM DTT, and 100 U of AcTEV protease (Invitrogen) at room temperature for 1 h. After centrifugation at 10,000×*g* for 10 min at 4°C, each supernatant was mixed with anti-FLAG M2 agarose (50 µl, Sigma-Aldrich) for secondary purification. The mixture was incubated at 4°C for 1 h, and after washing the precipitates with the wash buffer, RNase MRP was eluted with FLAG peptide in Tris-buffered saline (TBS: 20 mM Tris-HCl, pH 7.4, 135 mM NaCl, and 0.2 mg/ml 3× Flag peptide (Sigma-Aldrich)). The yield of the enzyme was 20 pmol from the 2-l yeast culture. The RNase MRP preparation thus obtained was used directly for the RNA cleavage assay. For the component analysis, the RNase MRP preparation was used after separation of RNA and proteins via phenol–chloroform extraction [82].

### Preparation of the core RNase MRP complex

To isolate the core of RNase MRP, the RNase MRP preparation on the anti-FLAG M2 agarose beads was incubated with 10 µg/ml RNase A (Sigma-Aldrich) at 4°C for 1 h. To eliminate RNase A completely from the complex, the beads were washed 10 times with vigorous agitation in 1 ml wash buffer (10 ml total, 200 volumes of the resin) before eluting the complex. The product of this partial nucleolysis was then eluted with FLAG peptide in TBS as described above and used for the *in vitro* RNA cleavage assay. The preparation thus obtained appeared free from RNase A activity, as the mock preparation obtained by the same procedure using RNase MRP without the tag did not cleave the RNA substrate, pre-tRNA^Ser-Met^. For the analysis of RNA and protein components, the eluate was extracted with phenol–chloroform, and the resulting water phase and organic phase were subjected to LC-MS/MS for RNA and protein analysis, respectively.

### *In vitro* RNA cleavage assay

RNA substrates were synthesized using an *in vitro* transcription T7 kit (Takara Bio). The DNA template for the transcription was made by PCR amplification from the *S. pombe* genome using the primers listed in Table S7. The RNase MRP or its core RNP complex (1 pmol) purified by immunoprecipitation was mixed with 1.25–16 pmol substrate in 20 µl digestion buffer (20 mM Tris-HCl, pH18.5, 10 mM MgCl_2_, 1 mM DTT, 100 mM KCl, 0.1 mg/ml BSA and 0.8 U/µl of RNasin (specific RNase inhibitor against RNases A, B, C, and placental RNase, Promega). We used this buffer solution to simulate the enzyme activity under the physiological condition, even though the activity might not be optimal for the cleavage of ITS1 sequence with respect to the potassium concentration [83]. After incubation at 37°C for 15 to 60 min, the reaction was stopped by adding water-saturated phenol. After ethanol precipitation of the aqueous phase, the digested substrates were separated by 8 M urea-7.5% PAGE and stained with SYBR Gold (Life Technologies). The profiles were scanned using a Fuji Film LAS-3000 Luminescent Image Analyzer and quantitated by Multi Gauge ver. 3.0 (Fuji film).

### Western blotting

Western blotting was performed using monoclonal anti-FLAG M2 (primary antibody, Sigma-Aldrich) and ECL anti-mouse IgG (secondary antibody, HRP-linked, species-specific whole antibody, GE Healthcare Life Sciences) diluted 1∶5000 in 5% skim milk in TBS-T (0.1% (w/v) Tween 20 in TBS). Peroxidase-conjugated monoclonal anti-HA (clone 12CA5, Roche Applied Science) was likewise diluted 1∶5000 with 2.5% skim milk in TBS-T. Chemiluminescence was initiated by staining with ECL Plus Western Blotting Detection Regent (GE Healthcare Life Sciences) and detected with the LAS-3000 Luminescent Image Analyzer.

### Ribonucleoproteomics procedures

Proteins were separated by SDS-PAGE on 15% polyacrylamide gels and in-gel digested as described [84]. LC-MS/MS was performed as described [82], [85]. A database search was performed using Mascot version 2.2.1 (Matrix Science) on the fission yeast protein dataset provided by the Wellcome Trust Sanger Institute (Spomb_20101102.fasta) using the search parameters described previously [82]. A peptide was considered “identified” if its probability-based Mowse score (total score) exceeded a predefined threshold that indicated significant sequence similarity (p<0.05). The threshold value was per the vendor's definitions (Matrix Science, Ltd.). Furthermore, we set a strict criterion that the overall sequence coverage of the identified peptides must exceed 40%.

RNAs were analyzed by LC-MS/MS directly without ethanol precipitation (for small RNA analysis), or after ethanol precipitation and urea-PAGE separation followed by in-gel RNase digestion (for large RNA analysis) [85]. RNases for in-gel digestion, RNase T1 (Worthington), MazF (Takara Bio), and PemK [86] were further purified before use [82]. The resulting RNA were analyzed by a direct nanoflow LC-MS/MS system as described [82]. The mass spectrometer (Thermo Fisher Scientific) was operated in a mode to automatically switch between Orbitrap-MS and linear ion trap–MS/MS acquisition as described. We used Ariadne software [57] for database searches for RNA. The database used was the genome sequence of *S. pombe* (http://www.pombase.org/downloads/datasets). The following search parameters were used: the maximum number of missed cleavages was set at 1; the variable modification parameters were two methylations per RNA fragment for any nucleotide; and an RNA mass tolerance of ±50 ppm and MS/MS tolerance of ±750 ppm were allowed.

### Determination of the stoichiometry of RNase MRP

The stoichiometry of protein components in the *S. pombe* RNase MRP was estimated by quantitative image analysis of SDS-PAGE profiles visualized by Coomassie Brilliant Blue R-250 staining. The profiles were scanned with a GT-X900 (Epson) and quantitated by Multi Gauge ver 3.0. The method provided a linear relationship between the signal intensity and protein quantity within the range of 0.1–1.0 µg protein as estimated with human serum albumin (data not shown). The quantity of each protein was expressed relative to Rmp1, which was given a value of 1.

### Affinity purification of ribosomal protein L7–associated proteins

Affinity purification of ribosomal protein L7-associated proteins (Rpl701, Rpl702, and Rpl703) was performed essentially as described under “Purification of the intact and core RNase MRP complexes” with minor modifications. Briefly, the transformants expressing the protein fused with a HATA tag were lysed, and each resulting cell lysate was incubated at 4°C for 1 h with human IgG–coupled Sepharose beads (GE Healthcare Bio-Sciences). The beads were washed with the wash buffer (50 mM HEPES, pH 7.4, 150 mM NaCl, 0.25% [v/v] NP-40) and incubated with the AcTEV protease–containing buffer at room temperature for 1 h. After centrifugation at 10,000×*g* for 10 min at 4°C, the supernatant was analyzed by Western blotting as described above.

## Supporting Information

Figure S1 **Illustration of synthetic tRNA substrates and their cleavage products with RNase MRP.** The synthetic tRNA mimics (pre-tRNA^Ser-Met^ and pre-tRNA^Ser^) and their cleavage products (trailer+tRNA^Met^ and tRNA^Met^) are indicated with their names and lengths (Table S7). The length of each component is indicated at the top of figure with an arrow. tRNA^Ser^ includes a 16-nt intron.
(TIFF)

Figure S2 ***In vitro*** **cleavage assay of an RNA fragment including ITS1 using** ***S. pombe*** **RNase MRP.** The purified RNase MRP was incubated with RNA including ITS1 (Table S7) at 37°C for 60 min, and the product RNAs were detected by 8 M urea-7.5% PAGE (SYBR Gold staining). Amounts (pmol) of RNase MRP and ITS1 used are indicated at the top.
(TIFF)

Figure S3 **MS/MS spectrum of the RNase T1 fragment of** ***mrp1*** **RNA with a trimethylguanosine cap.** The 5′ end of the RNase T1 fragment of the *mrp1* RNA (m3GpppAAAUGp^2−^, m/z = 1100.63) was analyzed by collision-induced dissociation. Observed fragment ions were assigned on the spectrum with an arrow (upper panel). The assigned ions were also expressed on the sequence with a bar (middle panel) and as the monoisotopic mass with red numerals (lower panel). Nomenclature of c- and y-series ions are according to Ni, J. et al. (1996) *Anal. Chem.*, **68**, 1989–1999. M, parent ion; p, phosphate; B, base; m_3_G, trimethylguanosine.
(TIFF)

Figure S4 **Detection of the interaction between RNase MRP and three Rpl7 isoforms (Rpl701, Rpl702, Rpl703).** HATA (HA, TEV cutting site, protein A)-tagged Rpl7 isoforms were expressed in JJ095 cells and pulled down with IgG-coupled Sepharose. The resulting precipitate was then analyzed by western blotting. Anti-FLAG was used to detect FEM-3-tagged Rmp1 in RNase MRP (upper panel), and anti-HA was used to detect Rpl7 isoforms (lower panel).
(TIFF)

Figure S5 **In vitro cleavage assay of an RNA fragment including ITS1 using RNase-resistant core MRP.** The RNase-resistant core MRP or intact RNase MRP (each 1 pmol) was incubated with RNA including ITS1 (1 pmol, Table S7) at 37°C for 60 min, and the product RNAs were detected by 8 M urea-7.5% PAGE (SYBR Gold staining).
(TIFF)

Figure S6 **SDS-PAGE profile of Rmp1-FEM3-tagged RNase MRP.** The Coomassie Blue–stained bands were cut into 15 pieces (Gel 1–15) and analyzed by LC-MS/MS for protein identification as described in Materials and Methods. The results of this analysis are given in Table S3.
(TIFF)

Table S1 Nomenclature of ribonuclease MRP complex subunits. Proteins in the same row are homologs. The components of RNase MRP and P shown here were identified in this study, predicted in Pombase, and reported by Dávila López M *et al*. and Esakova O *et al*. (*RNA Biol.* 2009; **6(3)**: 208–220. and *RNA.* 2010; **16(9)**: 1725–1747, respectively).
(XLSX)

Table S2 Summary of MS analysis of *mrp1* RNA isolated from *S. pombe* RNase MRP.
(XLSX)

Table S3 List of the proteins in *S. pombe* RNase MRP idenitified by the proteomics analysis.
(XLSX)

Table S4 Stoichiometory in the holoenzyme and core RNase MRP complexes purified from *S. pombe*.
(XLSX)

Table S5 The RNase A-resistant *mrp1* RNA sequence in RNase MRP identified by direct LC-MS analysis.
(XLSX)

Table S6 *S. pombe* strains used in this study.
(XLSX)

Table S7 Oligonucleotides used in this study.
(XLSX)
