# Supplementary figures and images for: RNase MRP Cleaves Pre-tRNASer-Met in the tRNA Maturation Pathway
Source: PLoS One. 2014 Nov 17;9(11):e112488. doi: 10.1371/journal.pone.0112488 (PMC4234475; doi:10.1371/journal.pone.0112488)

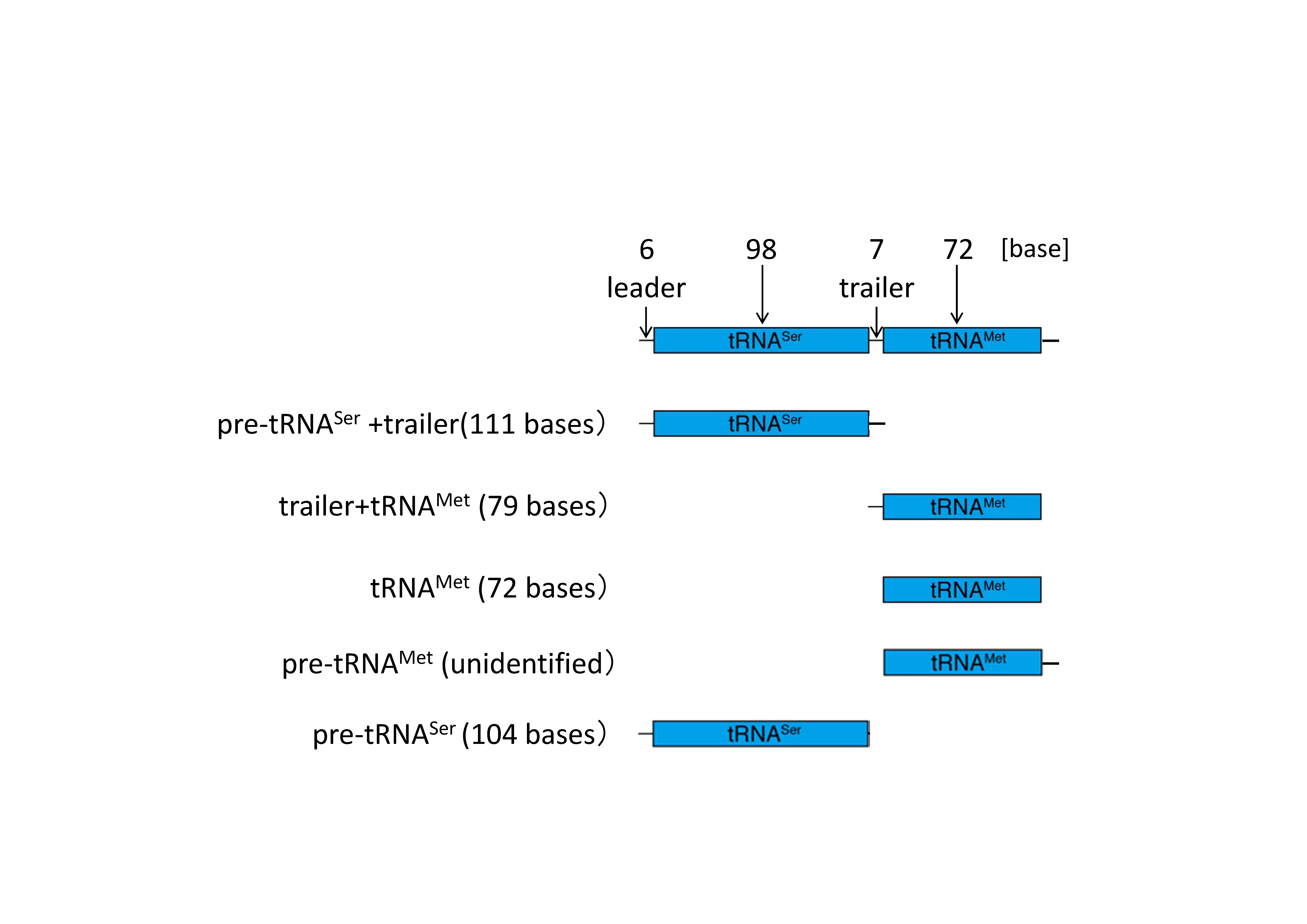

Supplement: Figure S1 — Illustration of synthetic tRNA substrates and their cleavage products with RNase MRP. The synthetic tRNA mimics (pre-tRNASer-Met and pre-tRNASer) and their cleavage products (trailer+tRNAMet and tRNAMet) are indicated with their names and lengths (Table S7). The length of each component is indicated at the top of figure with an arrow. tRNASer includes a 16-nt intron. (TIFF) [file pone.0112488.s001.tiff]

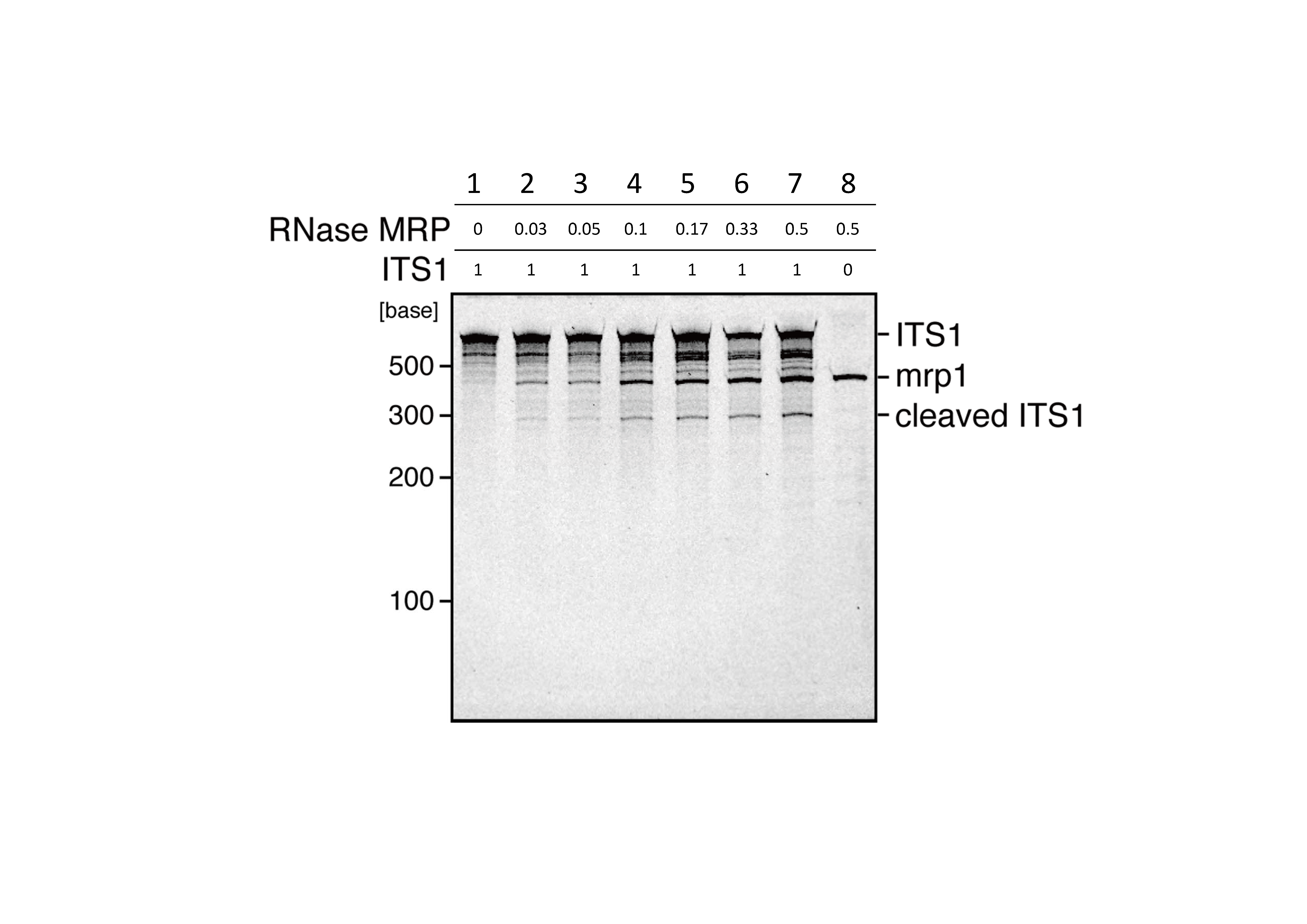

Supplement: Figure S2 — In vitro cleavage assay of an RNA fragment including ITS1 using S. pombe RNase MRP. The purified RNase MRP was incubated with RNA including ITS1 (Table S7) at 37°C for 60 min, and the product RNAs were detected by 8 M urea-7.5% PAGE (SYBR Gold staining). Amounts (pmol) of RNase MRP and ITS1 used are indicated at the top. (TIFF) [file pone.0112488.s002.tiff]

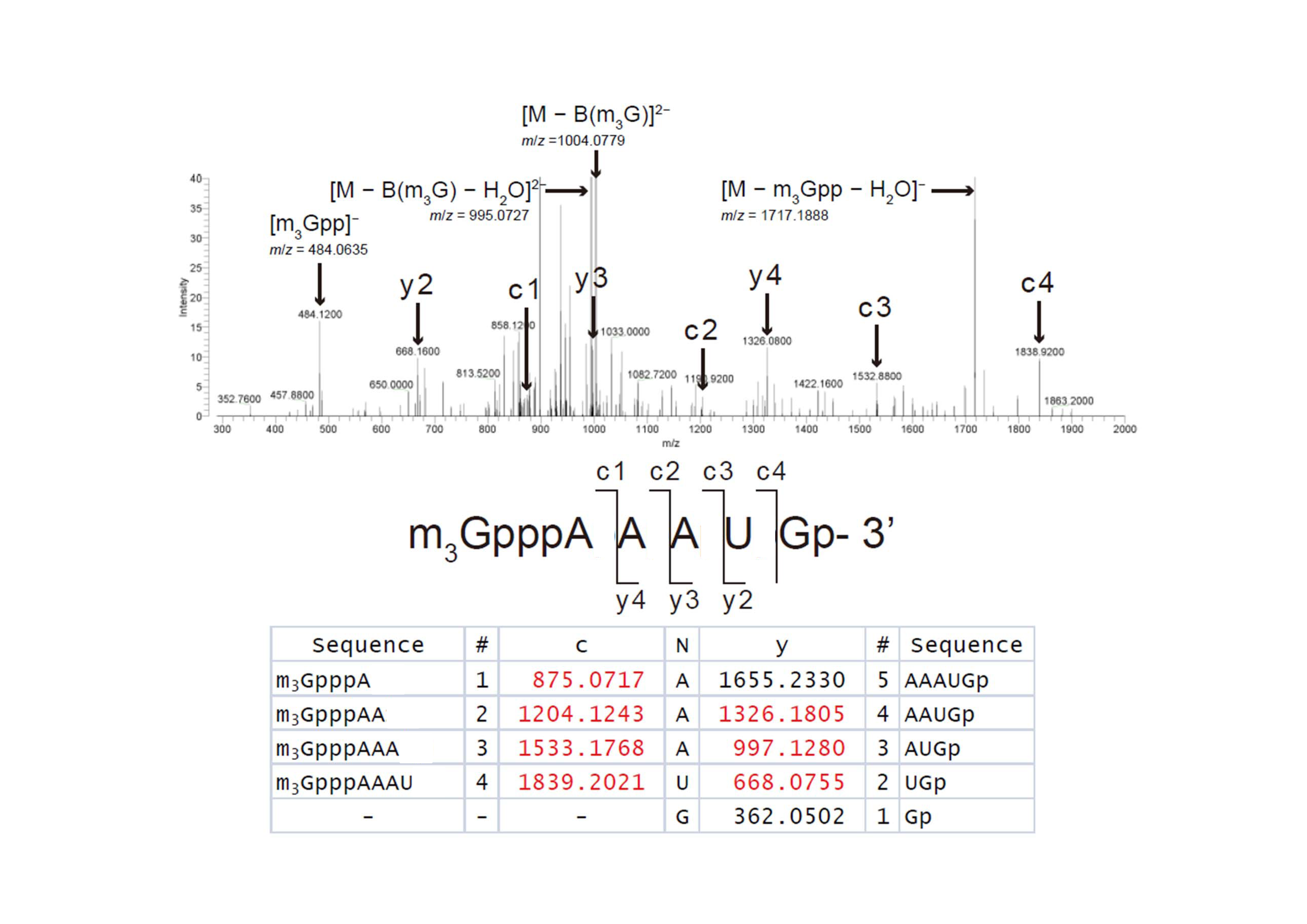

Supplement: Figure S3 — MS/MS spectrum of the RNase T1 fragment of mrp1 RNA with a trimethylguanosine cap. The 5′ end of the RNase T1 fragment of the mrp1 RNA (m3GpppAAAUGp2−, m/z = 1100.63) was analyzed by collision-induced dissociation. Observed fragment ions were assigned on the spectrum with an arrow (upper panel). The assigned ions were also expressed on the sequence with a bar (middle panel) and as the monoisotopic mass with red numerals (lower panel). Nomenclature of c- and y-series ions are according to Ni, J. et al. (1996) Anal. Chem., 68, 1989–1999. M, parent ion; p, phosphate; B, base; m3G, trimethylguanosine. (TIFF) [file pone.0112488.s003.tiff]

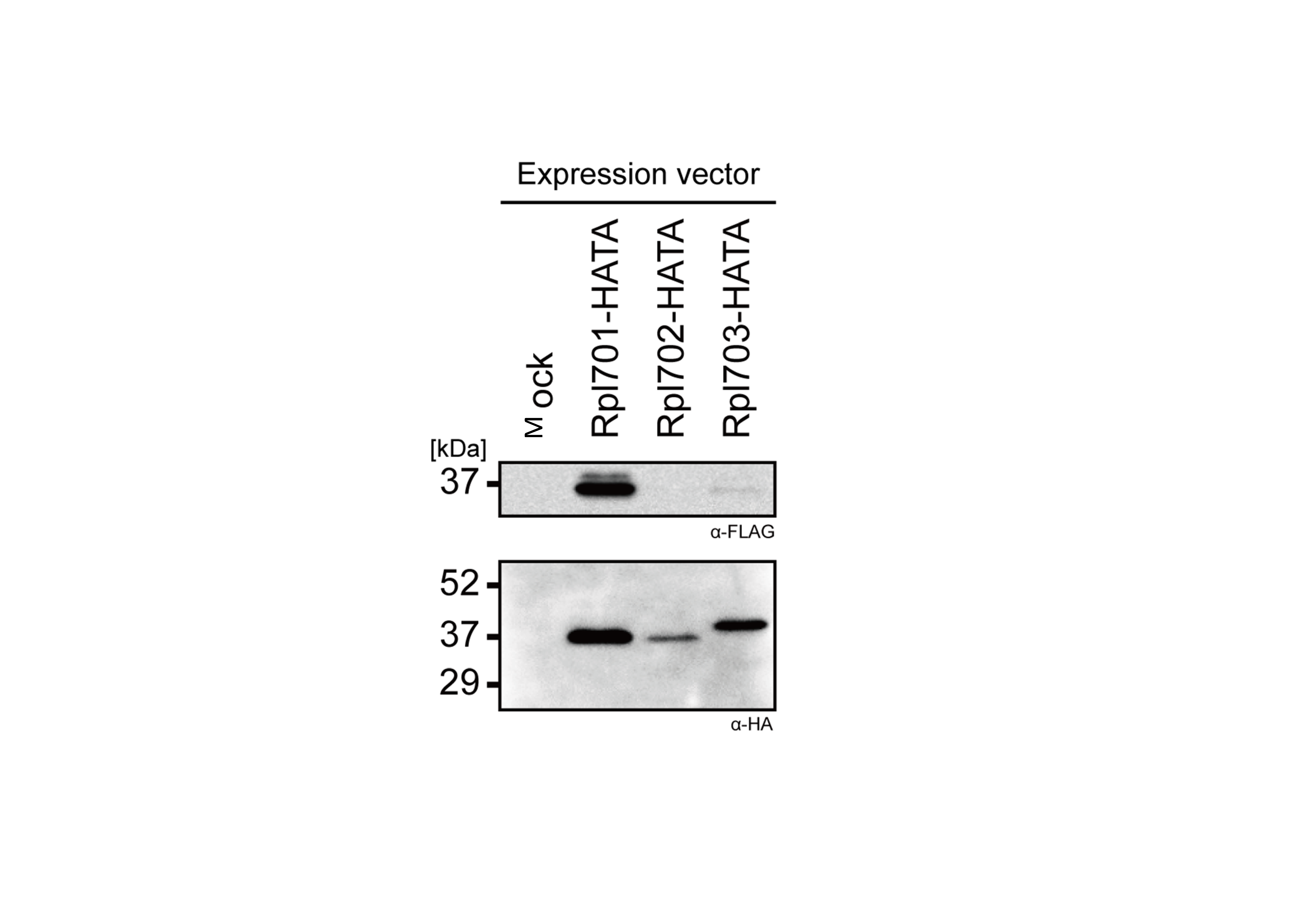

Supplement: Figure S4 — Detection of the interaction between RNase MRP and three Rpl7 isoforms (Rpl701, Rpl702, Rpl703). HATA (HA, TEV cutting site, protein A)-tagged Rpl7 isoforms were expressed in JJ095 cells and pulled down with IgG-coupled Sepharose. The resulting precipitate was then analyzed by western blotting. Anti-FLAG was used to detect FEM-3-tagged Rmp1 in RNase MRP (upper panel), and anti-HA was used to detect Rpl7 isoforms (lower panel). (TIFF) [file pone.0112488.s004.tiff]

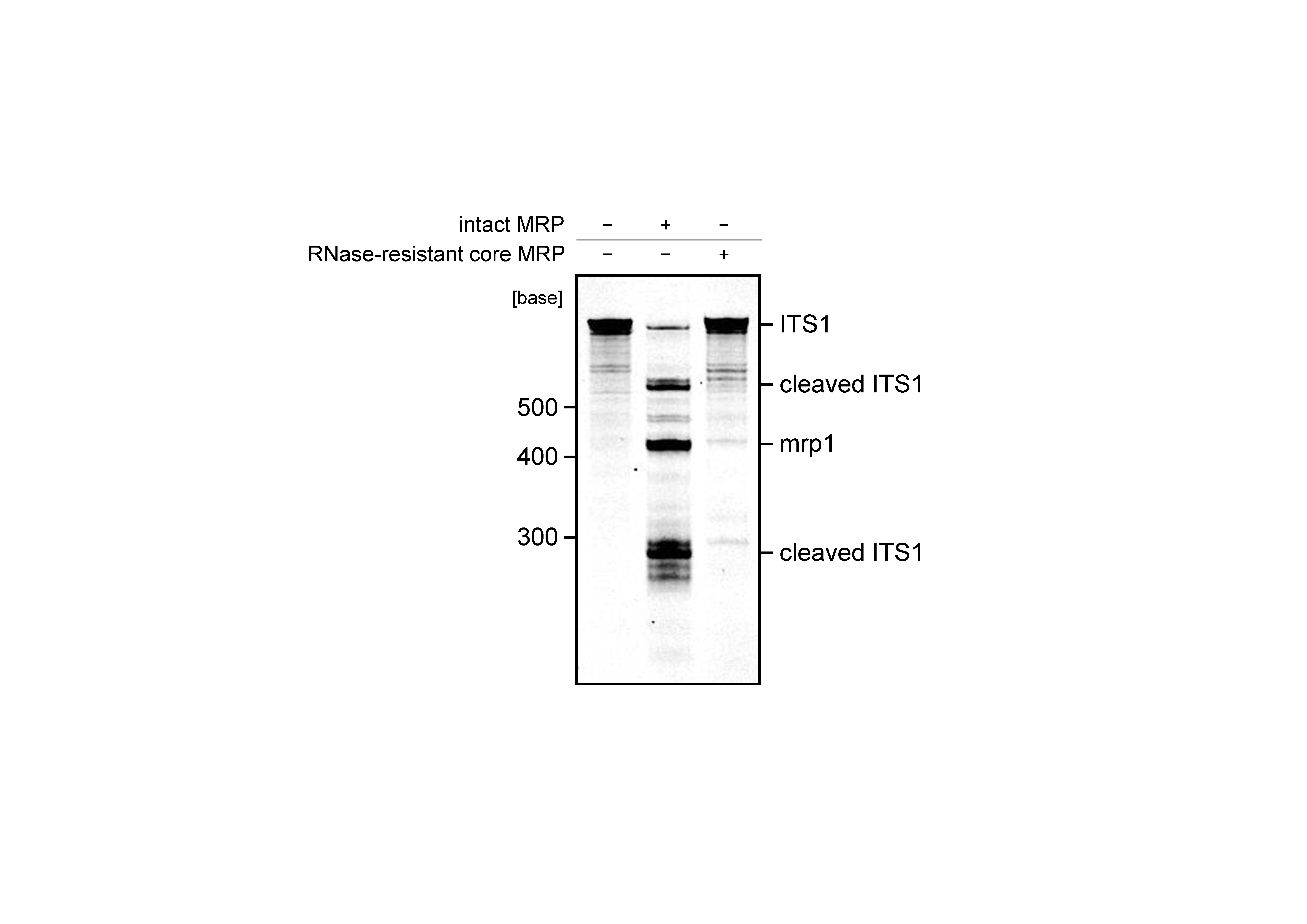

Supplement: Figure S5 — In vitro cleavage assay of an RNA fragment including ITS1 using RNase-resistant core MRP. The RNase-resistant core MRP or intact RNase MRP (each 1 pmol) was incubated with RNA including ITS1 (1 pmol, Table S7) at 37°C for 60 min, and the product RNAs were detected by 8 M urea-7.5% PAGE (SYBR Gold staining). (TIFF) [file pone.0112488.s005.tiff]

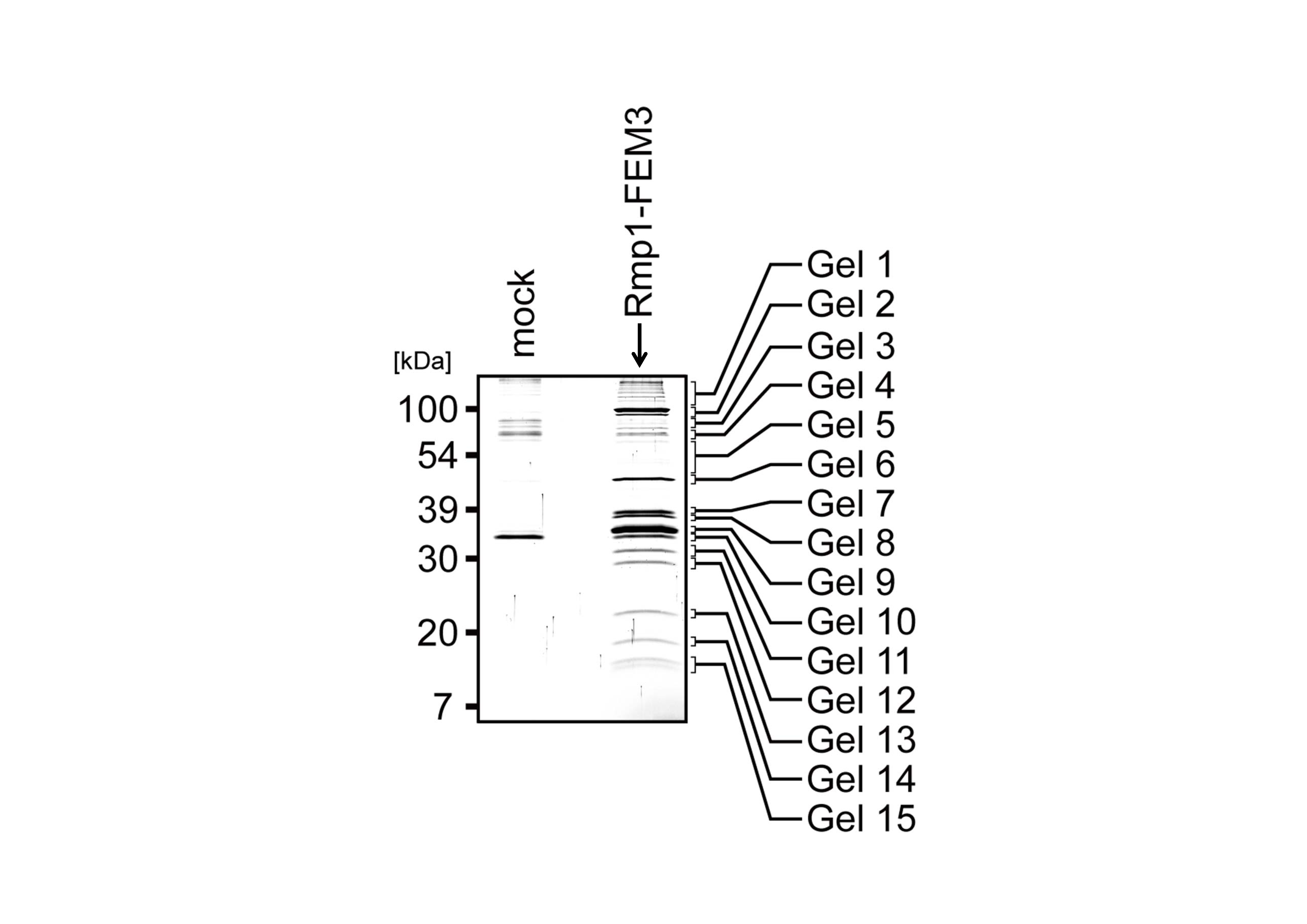

Supplement: Figure S6 — SDS-PAGE profile of Rmp1-FEM3-tagged RNase MRP. The Coomassie Blue–stained bands were cut into 15 pieces (Gel 1–15) and analyzed by LC-MS/MS for protein identification as described in Materials and Methods. The results of this analysis are given in Table S3. (TIFF) [file pone.0112488.s006.tiff]
